# Managing the Uncertainty and Accuracy of Life Cycle Assessment Results for the Process of Beverage Bottle Moulding

**DOI:** 10.3390/polym12061320

**Published:** 2020-06-10

**Authors:** Patrycja Bałdowska-Witos, Katarzyna Piotrowska, Weronika Kruszelnicka, Marek Błaszczak, Andrzej Tomporowski, Marek Opielak, Robert Kasner, Józef Flizikowski

**Affiliations:** 1 Department of Technical Systems Engineering, Faculty of Mechanical Engineering, University of Science and Technology in Bydgoszcz, 85-796 Bydgoszcz, Poland; weronika.kruszelnicka@utp.edu.pl (W.K.); a.tomporowski@utp.edu.pl (A.T.); robert.kasner@gmail.com (R.K.); fliz@utp.edu.pl (J.F.); 2 Faculty of Mechanical Engineering, Lublin University of Technology, 20-618 Lublin, Poland; k.piotrowska@pollub.pl (K.P.); m.blaszczak@pollub.pl (M.B.); m.opielak@pollub.pl (M.O.)

**Keywords:** PLA bottle, bio-based and biodegradable polymers, life cycle assessment, environmental impact, Monte Carlo

## Abstract

Using environmentally friendly materials in the technological process of bottle production fits perfectly into the idea of sustainable development. The use of natural raw materials as well as conscious energy consumption are strategic aspects that should be considered in order to improve the effectiveness of the bottle moulding process. This paper presents a new and structured approach to the analysis of uncertainty and sensitivity in life cycle assessment, one developed in order to support the design process of environmentally friendly food packaging materials. With regard to this “probabilistic” approach to life cycle assessment, results are expressed as ranges of environmental impacts, and alternative solutions are developed while offering the concept of input uncertainty and the effect thereof on the final result. This approach includes: (1) the evaluation of the quality of inputs (represented by the origin matrix); (2) the reliability of results and (3) the uncertainty of results (the Monte Carlo method). The use of the methodology is illustrated based on an experiment conducted with real data from the technological process of bottle production. The results provide insight into the uncertainty of life cycle assessment indicators regarding global warming, acidification and the use of arable fields and farmland.

## 1. Introduction

The point of reference used in the uncertainty assessment of life cycle assessment (LCA) results is the environmental evaluation of a life cycle, treated as a certain measurement technique based on a given methodology [1]. Its application leads to the “measurement” of two basic elements: environmental aspects that occur in the life cycles of products and subsequent environmental impacts [1,2,3,4].

Never can a single value without an uncertainty range represent the true value of an environmental impact because each measurement has uncertainty [5,6]. The process of producing bottles made of polylactide adopted in this paper focuses on the assessment of six technological operations, and concerns primarily the consumption of electricity, water and CO_2_ emissions. The implementation of biodegradable polymers into the production process is the result of increasing amounts of residual polymer waste. For years, plastics were produced to obtain durable, environmentally sensitive products. A change in strategy seeking alternative sources of materials has resulted in the development of biodegradable plastics. Therefore, research based on the LCA technique, presenting the potential size of environmental impacts, should also include the impact of uncertainty and the variability of results [7]. This approach is proposed by Lloyd and Riesa [8] at the stage of modeling the uncertainty of characterization test results.

Parameter uncertainty refers to the value of a parameter such as energy or raw material found in processes or products. In contrast, the uncertainty of the model refers to the specific model used, such as the model developed by ReCiPe for life cycle impact assessment (LCIA) by converting emissions and the extraction of resources into a limited number of environmental impact assessments using so-called characterizing factors [9].

The uncertainty analysis of life cycle inventory (LCI) data sets can be divided into quantitative and semi-quantitative analysis. The first is based on statistical methods for the quantitative uncertainty assessment of the LCI database [10,11,12,13], and the second on the data quality indicator (DQI) method [9,14,15,16,17].

Among the methods of semi-quantitative uncertainty analysis, it is worth considering the DQI semi-quantitative approach based on a qualitative assessment of data quality due to its widespread use in the field of LCA. The DQI approach can be divided into qualitative and semi-quantitative approaches. A qualitative approach evaluates data quality in terms of qualitative descriptors, such as good, fair and poor data quality, which depends on a rather subjective assessment. Usually, the method is subject to a qualitative approach [18,19]. The semi-quantitative approach adopts a numerical rating system, and the assigned quality rating is processed to obtain a single data quality score based on a probability distribution [20,21].

Sonnemann et al. [22] conducted a quantitative analysis of data uncertainty based on the Monte Carlo simulation, indicating its positive cognitive features. Maurice et al. [12] pointed out that quantitative uncertainty analysis is too time consuming. Lloyd and Ries, however, concluded that current quantitative uncertainty analysis does not address significant factors contributing to the uncertainty of LCA results due to the complexity of LCA models [8]. In their research, they also highlighted the problem associated with the long-term and time-consuming process of collecting input data from outside the data library, for example, Ecoinvent available in specific software, e.g., SimaPro or Gabi, which contributes to frequent references to literature. This is one of the reasons why semi-quantitative uncertainty analysis has been used in many studies [9,14,17].

The term uncertainty has a number of interpretations, including those that exclude related terms such as variability and sensitivity [23]. According to the international dictionary of metrological terms, uncertainty is a parameter associated with a measurement result characterizing the scatter of values which can reasonably be attributed to the measured quantity [24,25]. Uncertainty is characterized by a scatter of values (interval size), within which it is possible to place the measured value with a satisfying probability [26,27]. In the case of LCA, uncertainty is contained in the results of “measurement” obtained at different research levels, that is, after the analysis of a set of entries and exits related to environmental aspects (LCI results) and after the environmental impact life cycle assessment (LCIA results) [28].

Considering the complexity of calculations, it is very difficult to present the uncertainty of LCA results in the form of a single equation describing the probability distribution of the values obtained. Therefore, in order to estimate these uncertainties, numeric simulations are conducted. B. Steen [29] indicates that sensitivity analysis enables one to express uncertainty in the form of probability, hence the need to estimate the degree of uncertainty and the distribution of probability. When analysing the LCA method, Kowalski and Kulczycka [30] recommend thorough sensitivity analysis or, if possible, partial uncertainty analysis of selected results and parameters whose uncertainty ranges are known, e.g., by means of the Monte Carlo (MC) simulation. He also indicates that some studies involving the use of LCA are subject to uncertainty, which can result in doubts regarding the value of final indicators, eco-indicators that determine the potential environmental impact of a product or process. The uncertainty of model correctness results from the fact that analysed models are never real. Every LCA is subject to uncertainty which is due to subjective choices made in order to design the model [31].

The existing literature on the theoretical foundations of the mathematical computational structure for performing uncertainty analysis in LCA is still hardly practiced [32]. However, there are various techniques for uncertainty analysis, such as: the theory of possibilities, e.g., [33], fuzzy theory, e.g., [34,35], data quality indicators, e.g., [9,14,15,16,17] and expert opinions, e.g., [36,37] or a combination of two or more techniques, e.g., [20,30,31,38]. Despite the wide range of approaches available, the analysis of uncertainty in LCA in the scientific and business process of bottle production remains largely unexplored.

Stochastic modeling, mainly in the form of MC simulations, is the most commonly used approach to analyzing uncertainty among various processes, research areas and industry sectors [39,40,41,42]. The usefulness of MC simulation becomes apparent when enough tests have been performed to make a claim regarding data distribution and uncertainty [30]. However, the combination of two elements of stochastic modeling and uncertainty analysis can be problematic. It most often lists three main reasons: (1) there is a need for a larger amount of data to be available than is available [43,44,45]; (2) there are no clearly described guidelines as to the size of the data set, and this extends the duration of the analysis and the complexity of calculations considerably [46]; (3) the choice of combined research methods seems to be too complex [47]. The most common in the literature are works carried out omitting accuracy analysis [48], or those using error propagation methods, including sampling techniques [49]; much less research is carried out using MC simulation.

The uncertainty of data can be expressed by means of the probability distribution thereof, e.g., the standard deviation or variance. This enables one to specify the range of values they can take. According to R. Heijugns [50], using the MC simulation in iterative LCA is time-consuming, but this disadvantage can be dealt with by calculating the propagation of uncertainty within the LCA methodology. H. Imbeault-Tétrault [51], when comparing the uncertainty propagation calculations with the MC simulation for uncertainty analyses within the LCA methodology, confirmed that the calculation of uncertainty propagation requires less computation time than the MC simulation and suggested that the analytical approach should be used instead. Heijugns and Lenzen [49] describe in detail where uncertainty can be encountered in LCA. MC is time- and cost-efficient, enabling one to determine the confidence level [52].

Uncertainty in studies involving the use of LCA can result in doubts regarding the value of final indicators (eco-indicators) that determine the potential environmental impact of a product or process. There are three types of uncertainty: uncertainty of data, uncertainty related to the correctness (representativeness) of the model applied and uncertainty caused by incompleteness of the model.

In the analysis of data uncertainty conducted by Lewandowska and Fołtynowicz [31], the basic assumption is that the quality of input data should increase with its importance. The authors propose analyzing data quality after having evaluated their environmental impact. As impact assessment is performed for the whole system within precisely specified boundaries, the final result pertains not only to the main data input, but also to the data of all the processes it represents.

Using statistical terms, LCA is used to study and compare systems of products in terms of the same feature, that is, environmental impact [53]. Uncertainty analysis, however, is reduced to determining the feature diversification level for each of the systems. The more diversified the feature is (scatter) the higher the uncertainty it represents [26,52,54].

This article aims to supplement the issues and knowledge about the environmental impact of the bottle shaping process presented in [55] by addressing the quality issues of the required data and the complexity of stochastic modeling for uncertainty and sensitivity analysis, thereby offering a new life cycle assessment model not yet practiced by food sector companies in Poland. The purpose of this paper, therefore, is to propose an evaluation method based on the use of the DQI semi-quantitative approach, stochastic modeling and sensitivity analysis to (1) analyze the uncertainty of beverage bottle production parameters, (2) analyze the uncertainty and accuracy of LCA results for the bottle shaping process and (3) recognize the effects of input parameters on the final results obtained.

## 2. Materials and Methods

### 2.1. The Uncertainty and Accuracy Management Procedure

Accuracy is defined as the similarity of the measured or modeled value to the “real one” [56]. Precision, however, represents the quality of repeatability of the results obtained, e.g., each repetition of calculation, of the experiment, and modeling provides a similar result [57,58].

Accuracy and precision are provided by methods which are independent of each other; in other words, a method is imprecise when the results exhibit a large scatter, yet it is accurate, since the mean value of the results corresponds to the real, model or theoretically predicted value [24]. A method is precise but not accurate when the scatter of the results is not large, but the mean value is far from the real, model or theoretically predicted value [59].

**Figure 1:**
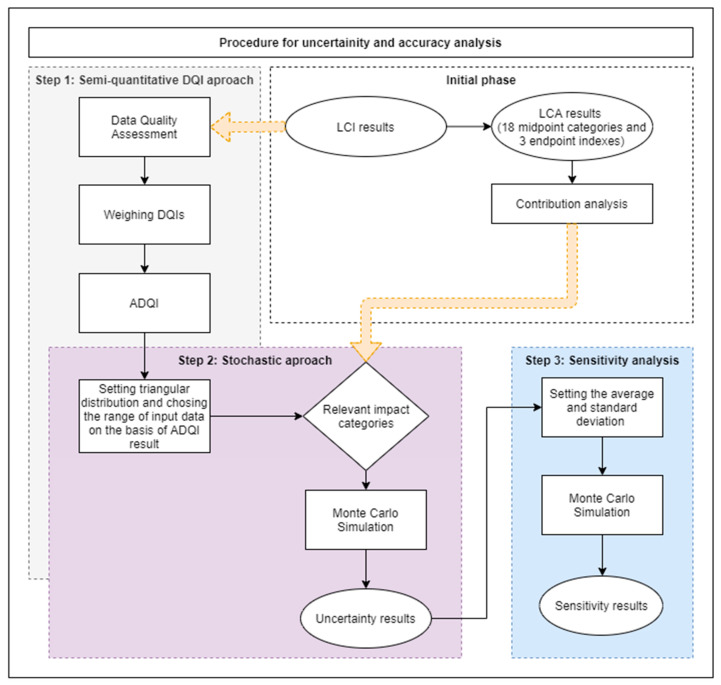
Flow chart of the uncertainty and accuracy management procedure.

In this paper, a procedure for estimating data uncertainty and accuracy based on the known methods for determining the quality and uncertainty of data is proposed. The method combines three approaches: the DQI semi-quantitative approach, the stochastic modeling approach and the global sensitivity analysis. The procedure includes (Figure 1):

- An initial phase, which consists of LCA with contribution analysis,
  Step 1: the semi-quantitative DQI approach,
  Step 2: the stochastic modeling approach with use the MC simulation,
  Step 3: the sensitivity analysis based on the analysis of key issues.

The steps of the procedure are closely related, in such a way that the results of the preceding step are input data for the following step. In this way, it is possible to determine the sensitive points of the LCA along with the determination of the relationships and dependencies of the input data uncertainty with the uncertainty of the results in terms of impact categories and damage areas.

The first initial phase is the LCA allowing the identification of process input data (LCI) and environmental impacts (LCIA). An indispensable element for the proposed procedure is the implementation of contribution analysis to determine the unit processes and categories of impacts with the largest share in the total impact of the PLA bottle shaping production cycle. The LCA of this process was presented in a previous work [55]. The uncertainty analysis involved input data and only relevant impact categories in damage areas. The categories with total contribution equal to 90% were considered relevant. A detailed LCA methodology is provided in Section 2.2.

The first step in assessing data uncertainty is the semi-quantitative DQI approach. It includes the standard quality assessment proposed by Maurice et al. [12] with the use of five DQIs [25,56]: measurement precision (reliability), sample representativeness, appropriate age of data, geographic origin and technological representativeness, and then calculating the aggregated data quality indicator (ADQI) and determining the deviation of input data. A triangular distribution for pro-ecological scenarios of a product life cycle assessment was used in the analysis. The information about distribution obtained in the previous step is used as an input to the second step of the stochastic approach to estimate the uncertainty of significant impact categories using MC simulations. In this way, the impact categories with the greatest uncertainty and the input data of the bottle shaping process that are associated with them, will be identified. As a result of MC simulation, statistical parameters such as average, standard deviation, variance and coefficient of variation are obtained for the distribution of results of significant impact categories. Thereby, it is possible to carry out the key issue analysis using the MC simulation, where the mean and standard deviation will be used as input data for forecasting the sensitivity of the results of the total impact of the bottle shaping process to changes in the impact category value, and thus related changes of inventory data. A detailed methodology of individual stages of the embedded method is presented in Section 2.1.1, Section 2.1.2 and Section 2.1.3.

#### 2.1.1. Semi-Quantitative DQI Approach

The qualitative data evaluation was carried out using data quality indicators (DQIs). In the analysis the pedigree matrix (Table 1) was used, which is determining five different DQIs: reliability, completeness, time range, geographical range and technological range [18]. First, the input data were assessed in qualitative terms and then in quantitative terms, assigning points on a scale of 1 to 5 for each DQI [25,56]. Depending on the methodology adopted, grade 1 may mean the highest rating (which is consistent with the general method of the matrix of origin [18] and appears in many works, e.g., Baek et al. [20,38], Maurice et al. [12], Ciroth et al. [52]), or, in line with the approach used, for instance, by Lewandowska [60], Canter et al. [15], Kennedy et al. [17], data of the highest quality receive a rating of 5. In this work, we assumed that a score of 1 indicates the lowest quality of data and 5—the highest. DQA was carried out at the level of the unit process, for which each input parameter had the same data source. The selected level of data quality assessment DQA is an attempt to apply the DQI semi-quantitative approach as a practical and simple means to analyze parameter uncertainty.

In the following approach, the so-called aggregated data quality indicator (ADQI), which is the sum of five weighted DQIs, was used [12]. Initially, the weight of each input parameter was obtained by assigning 1 to 5 points to each of the five DQIs. To determine the ADQI value, it is necessary to assume the weight for each DQI. In the simplest terms, it can be assumed that individual DQIs are equivalent. In many works, however, there are methods for determining the weight of individual indicators [12,14,20]. In this work, we adopted weights according to Maurice et al. [12]: 0.25 in relation to the geographical and technological scope and 0.167 in relation to the other three DQIs. Considering the input data of the bottle shaping process (mainly—electricity consumption), it is the geographical and technological scope that will cause the greatest diversity of data, and thus affect the reliability and credibility of LCA results, so it was decided to take the weights as proposed by Maurice et al. [12]. Next, the DQI value was multiplied by the weight of each DQI and the resulting values were summed up.

The next stage of the DQA is to relate the results obtained from origin matrixes to probability distributions [14,17,60]. In the case of point data or very small data sets, reliable determination of the average or standard deviation is not possible [60]. Then, it is necessary to use distributions based on other parameters, e.g., triangular distributions, continuous uniform or beta distributions [17,20]. Triangular and continuous uniform distributions are based on two parameters: minimum and maximum values [60].

**Figure 2:**
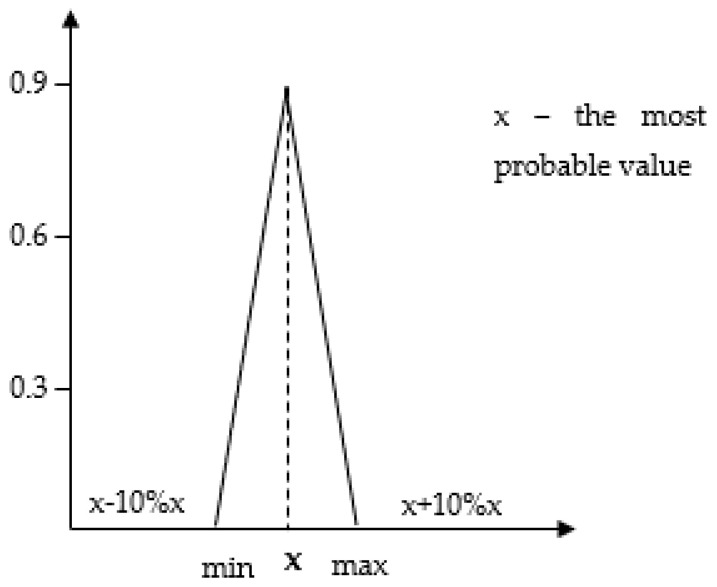
Dependence between the ADQI value and the shape of the triangular distribution curve in the process of polylactic acid bottle manufacturing.

Attempts to link DQA results to the distribution of input data were made by Kennedy [17] and Wang [14] using the so-called transformation matrix. In this work, we combined the ADQI results with the levels of deviations of the process input values (Table 2) assuming a triangular distribution. Therefore, it was assumed that the data whose DQI score is 5 has a permissible standard deviation of ±10%. As can be seen, a deviation from the most probable value occurs only at the level of ±10%, which is manifested by a narrow triangular distribution (strong concentration around the central value) and high (high probability) triangular distribution shape. The idea of triangular distribution is explained in Figure 2. This means that a person who performs an LCA assumes that the value of a given inventory (Table 3) (e.g., energy consumption, water consumption, CO_2_ emissions) does not necessarily have to be x value (the result of a single measurement, estimation, collection from the company documentation, etc.), but that it can be a value from the range < x − 10% x; x + 10% x >.

The deviation ranges obtained on the basis of the ADQI values and the selected data distribution will be the input data in the next step of the stochastic uncertainty analysis approach using MC simulations, which will be described in detail in Section 2.1.2.

#### 2.1.2. Stochastic Modeling Approach

The approach to stochastic modeling using MC simulation is a common method of quantitative uncertainty analysis in LCA. In this work, we used the MC simulation, because it allows one to quickly estimate the probability of results depending on the variability of input data, and thus identify the results with the highest sensitivity and uncertainty. The first assessment step is to correctly define the input parameters necessary to conduct the analysis properly. Triangular distributions based on two values—maximum and minimum—were used to estimate the impact category uncertainty. At this stage, the influence of the input parameters on the significant impact categories (impact categories which in total constituted 90% of damage area were considered as significant) was estimated. According to the adopted procedure, the values of the input parameter deviations depend on the ADQI result according to Table 2. For each input parameter, a value within the range of data variability was artificially generated and then values of selected impact categories were calculated. The procedure was repeated 1000 times to obtain the uncertainty distribution. This simulation was carried out using Sima Pro software.

#### 2.1.3. Sensitivity Analysis

The correct interpretation of LCA results should assess the reliability of data and the uncertainty of results. For this purpose, among others, contribution analysis, perturbation analysis or sensitivity analysis are used [61]. Based on the sensitivity analysis, the usefulness of individual data can be inferred by indicating key variables that cannot be omitted in the analysis [62]. Performing a sensitivity analysis is considered to be one of the good practices in LCA [63,64]. There are three main approaches to sensitivity analysis mentioned in the literature: local, screening and global [62,65,66]. Local sensitivity analysis includes matrix perturbation and the once-at-a-time method. The method of elementary effects is a screening method designed by Morris and can be treated as an extension of the once-at-a-time method [66]. Global sensitivity analysis is mainly based on the analysis of variance of input variables. Among the methods of global sensitivity analysis, LCA uses the key issue analysis, the method of standardized regression coefficients, and Sobol sensitivity index [65].

In this work, the global sensitivity analysis method was used, namely the key issue analysis introduced by Heijungs [67,68]. This method is based on the analysis of the contribution of variables in variance and allows to determine the share of the input data uncertainty in the result uncertainty [68]. An analysis of data sensitivity was carried out for data which were in impact categories characterizing the overall impact of the PLA bottle shaping process in three areas: human health, ecosystem quality and resource avaliability. The procedure was carried out taking into account the variability of the analyzed parameters, using the MC simulation and the Crystal Ball (CB) software. The sensitivity analysis was presented in three formats: the grouped bar chart, the tornado type chart and the spider chart.

In the MC simulation the results obtained on the basis of LCA were used. The log-normal distribution, which is commonly used in analyzing data uncertainty, was used to estimate the value of the impact category in the Monte Carlo method [20,62]. Distribution parameters, i.e., population mean µ and standard deviation σ were adopted respectively: µ as the mean value of the impact category obtained as a result of data uncertainty analysis, σ as the standard deviation of the impact category obtained as a result of data uncertainty analysis. Tornado and spider charts were created based on the results of MC simulations. Relevant impact categories in LCA were used to generate charts.

### 2.2. Goal and Scope

The first step in LCA is to specify the objective and scope of the analysis, which can be determined based on the analysis and understanding of the product lifecycle. The research objective determines the degree of detail, thoroughness and scope of analyses, as well as the types of data needed to evaluate the lifecycle. To this end, the technological process of biodegradable polylactic acid (PLA) bottles shaping was subjected to evaluation. The process was broken down into six-unit operations, taking into account the demand for services and materials. The scope of the analysis covered the intake of pre-moulds into the heater, the heating of said moulds in the infrared heater, the stretching, extending, and pressure shaping of the pre-mould, as well as degassing and cooling the moulded bottles. The environmental impact of the bottle moulding process was performed using the ReCiPe 2016 method. The analysis covered 17 midpoint impact categories and three endpoint damage categories: human health, ecosystem quality and resource availability, which strictly correspond with three areas of protection: human health, ecosystem quality and resource scarcity [69,70]. Characterization factors from the endpoint level were obtained from the midpoint characterization factor using the constant midpoint to endpoint conversion factor [70,71]. The human health endpoint category includes impacts from the following midpoint level categories: particulate matter, trop. ozone formation (hum), ionizing radiation, ozone depletion, human toxicity (cancer), human toxicity (non-cancer), global warming, water use. The ecosystem quality endpoint category includes global warming, water use, freshwater ecotoxicity, freshwater eutrophication, trop. ozone (eco), terrestrial ecotoxicity, terrestrial acidification, land use/transformation, marine ecotoxicity. Finally, the resource availability category includes mineral resources and fossil resources. The LCA analysis was performed using the SimaPro software. Thereafter, relevant midpoint and endpoint categories were chosen. The categories with total contribution equal to 90% were considered as relevant. The aim of this analysis is to assess the uncertainty and accuracy of LCA results using the Monte Carlo method. The basis for looking for key factors was the uncertainty analysis of key results for which statistical and mathematical methods were used.

#### 2.2.1. Functional Unit

The functional unit accepted for the analyses was determined based on data collected from the production plant. A 1 L PLA bottle was adopted as the functional unit.

#### 2.2.2. System Boundaries

**Figure 3:**
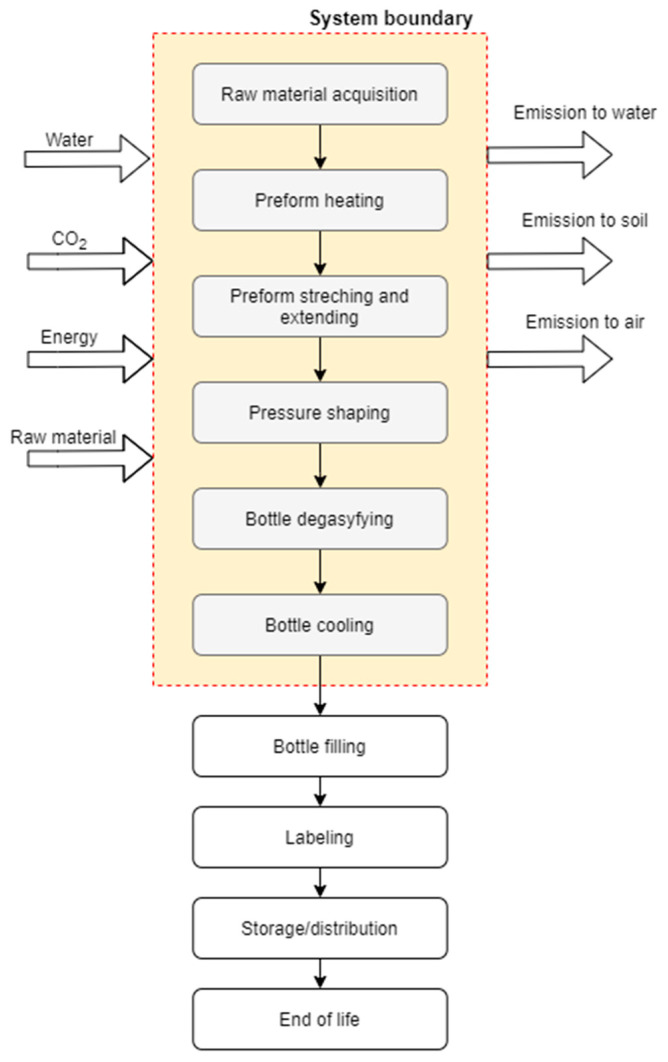
System boundaries for the environmental analysis of the polylactic acid (PLA) bottle production process.

The analysis covers the entire cycle of the bottle shaping process, meaning that that all steps of this process are included, from the pre-mould delivery to the manufacturing company, up to the moment when beverage bottles are correctly shaped in the moulding process (Figure 3). Further processes, such as the filling of bottles with beverages, the labelling or storage/distribution thereof were not included in the system. Furthermore, the transportation of raw material and storage was also omitted.

#### 2.2.3. Data Allocation

The allocation procedure is described in detail in ISO 14044 (clause 4.3.4. Allocation, Section 4.3.4.2 Allocation procedure) [2]. It is particularly important when considering multifunctional processes. For this reason, allocation, understood as the partitioning and attribution of environmental pressures to products/functions of the analysed system, is one of the most frequently applied multifunctional solutions. In the case of bottle moulding, the technological process was divided into smaller technological operations. Such a procedure does not require any partitioning; therefore allocation is not required.

### 2.3. Life Cycle Inventory

Designing a model for the analysis of the set of inputs and outputs is the second phase of LCA. The model reflects the whole product system, while its smaller elements represent technological operations. A technological operation should be understood as the smallest part of the system for which resource-related information is collected. Data collecting enables one to precisely specify the source of origin, geographic scope, representativeness and precision, all of these being indispensable elements of the uncertainty analysis [56]. The correct aggregation of input data helps to identify significant environmental points in the system.

Taking into account the confidential character of the LCI results presented in the study and company trade secrets, the values presented in Table 4 were changed by a coefficient ranging from 0.8 to 1.2. The data describing all of the process stages come from one company in Poland and pertain to the bottle moulding process taking place there. The figures apply to the year 2019. The modelling was performed using the Ecoinvent 3.2 database.

## 3. Results

### 3.1. Contribution Analysis

**Figure 4:**
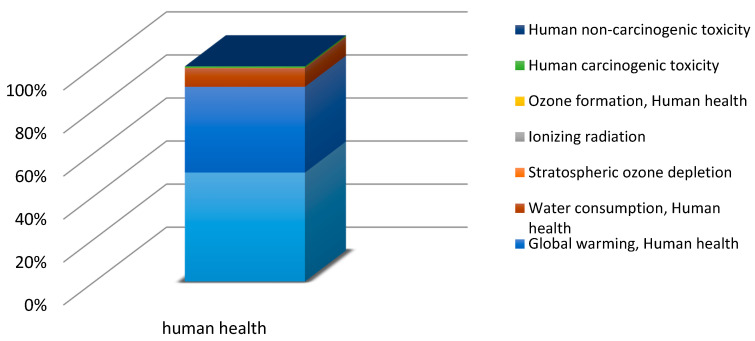
Contribution analysis in the human health damage category.

**Figure 5:**
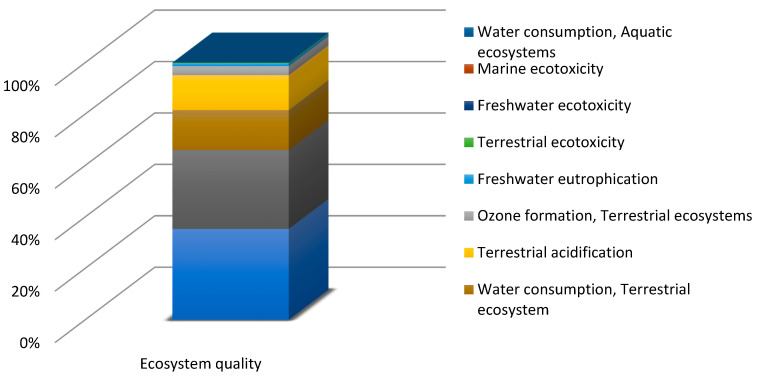
Contribution analysis in the ecosystem quality damage category.

**Figure 6:**
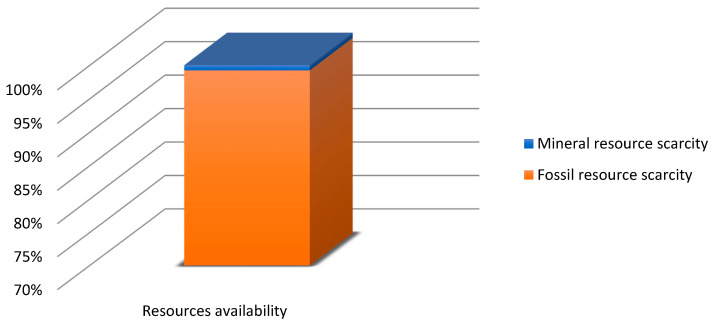
Contribution analysis in the resource availability damage category.

Before commencing the uncertainty analysis, it is necessary to obtain LCA results. This paper analyses the quality, uncertainty and sensitivity of data in the LCA of the PLA bottle moulding process. [55] presents a complete set of the system characterisation results. The uncertainty analyses were performed using relevant impact categories only (with the summarised share of 90% at least). In the human health area, relevant impact categories included (Figure 4): fine particulate matter formation (50.68%), global warming human health (39.82%), water consumption human health (8.64%). As regards the ecosystem quality area, these were (Figure 5): global warming terrestrial ecosystems (35.31%), land use (30.77%) and water consumption, terrestrial acidification (15.44%), terrestrial acidification (13.59%). For the resource availability (Figure 6), the fossil resources scarcity category was significant (99.23%). The pre-form material and the electric energy consumption in the bottle moulding process were the most important determinants of the above-listed categories value.

**Figure 7:**
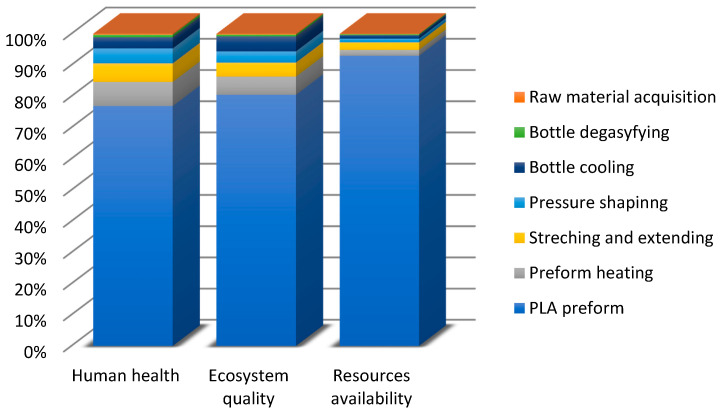
Contribution analysis of the operation of bottle shaping process in damage categories.

In the damage category, the impact of pre-form material—PLA in this case—prevailed (Figure 7). In the category of human health, the impact of pre-mould (76.86%) was followed by the pre-form heating phase (7.72%) and the stretching and extending stages (5.93%), while in the ecosystem quality category, the role of pre-form heating (5.81) and bottle cooling (4.79%) can be noticed.

### 3.2. Semi-Quantitative DQI Aproach

For the first stage of the data quality and uncertainty assessment, the semi-quantitative DQI approach was adopted. The DQI and ADQI values were determined according to the procedure described in Section 2.1.1. A summary of results is presented in Table 5. The input data for each DQI were assigned 5 points, as they were obtained from measurements taken within one company and one process and were collected over a period of one year. Therefore, with such a high precision, data can be expected to provide reliable final results of the PLA bottle moulding process analysis. Following the multiplication of DQI by an appropriate weight coefficient, ADQI = 5 was obtained. Based on the ADQI value and Table 3, the deviation for each input value was determined. Using triangular distributions, the value provided in the inventory analysis is treated as the most probable value, and knowing its ADQI score, it is possible to define the minimum and maximum of input data.

The data presented in Table 5 are the results of the estimated minimum and maximum values for the processes involved in PLA bottle shaping. According to the accepted minimum and maximum thresholds, it can be said that the value of energy consumption for the first operation ranges from +0.330 to +0.404.

### 3.3. Uncertainty Results

**Figure 8:**
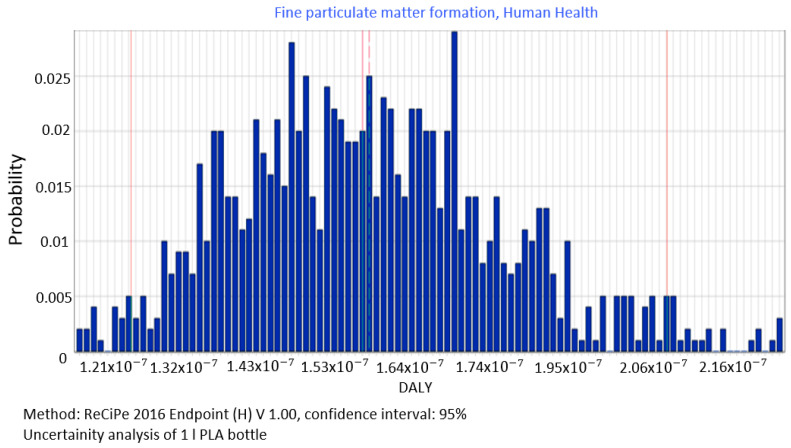
Results of the uncertainty analysis for the category of the formation of fine solid particles and their impact on human health.

Using the function of uncertainty 1000 values of impact category on the basis of the MC analysis were generated for each entry and exit, according to the information about variability ranges of input parameters and their distribution (in this study triangular) presented in Section 3.2. During the simulation, values of input parameters such as: water, electrical energy use, material consumption and emissions are randomly selected for calculation of impact assessment. On this basis, distribution histograms of the selected relevant impact category results (Figure 3, Figure 4, Figure 5, Figure 6, Figure 7 and Figure 8) were created for the randomly selected values of the input parameters. The analysis also provides basic parameters from the set of results obtained, such as standard deviation, mean, median and the coefficient of variation.

**Figure 9:**
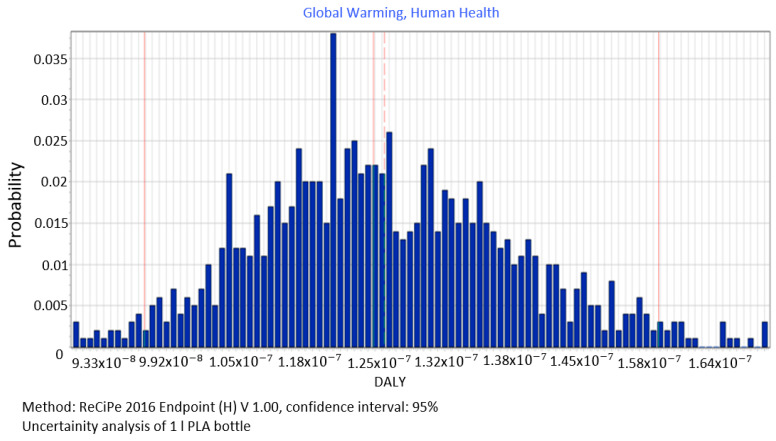
Results of the uncertainty analysis for the category of global warming and its impact on human health.

**Figure 10:**
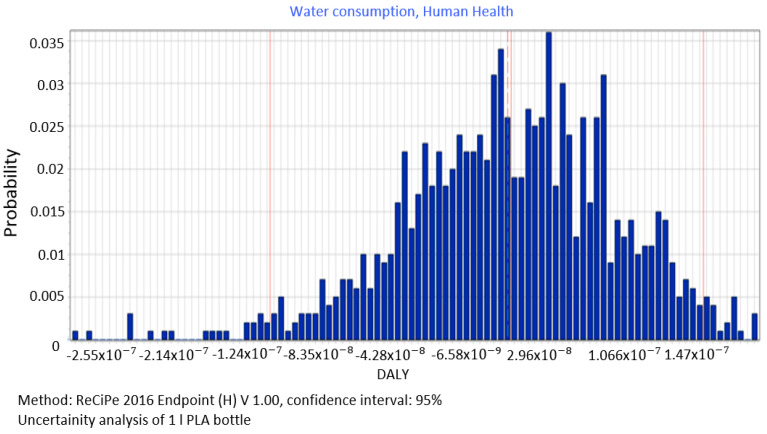
Results of the uncertainty analysis for the category of water consumption and its impact on human health.

**Figure 11:**
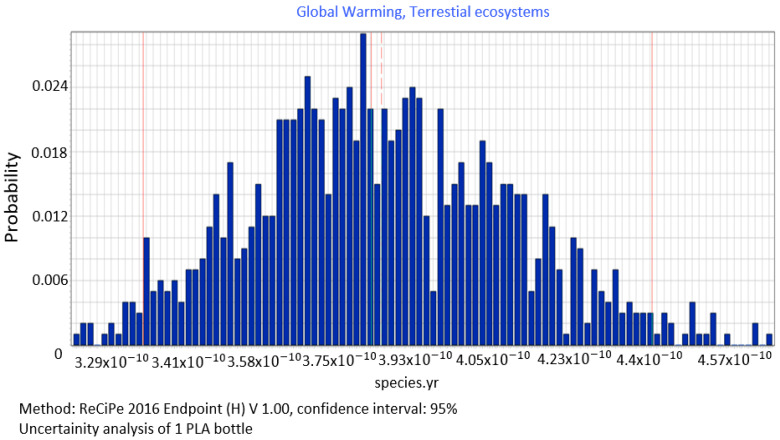
Results of the uncertainty analysis for the category of global warming and its influence on the ecosystem quality.

**Figure 12:**
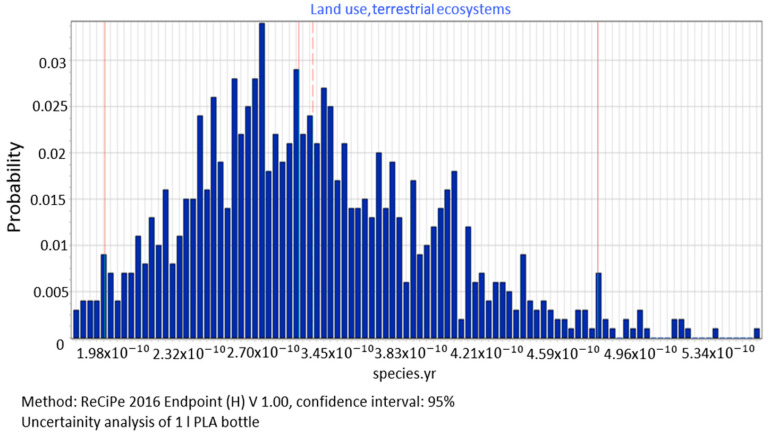
Results of the uncertainty analysis for the category of land use and its impact on the ecosystem quality.

**Figure 13:**
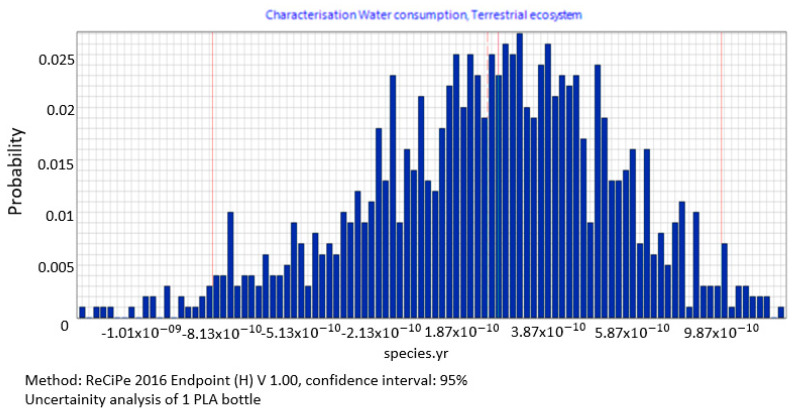
Results of the uncertainty analysis for the category of water consumption, terrestrial ecosystems and its impact on the ecosystem quality.

**Figure 14:**
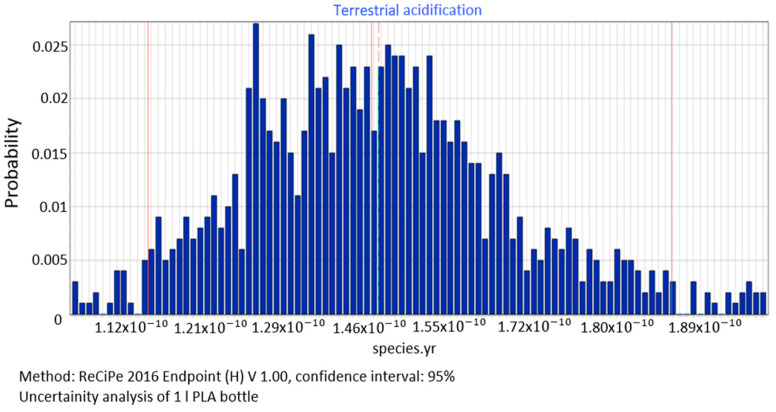
Results of the uncertainty analysis for the category of acidification and its impact on the ecosystem quality.

**Figure 15:**
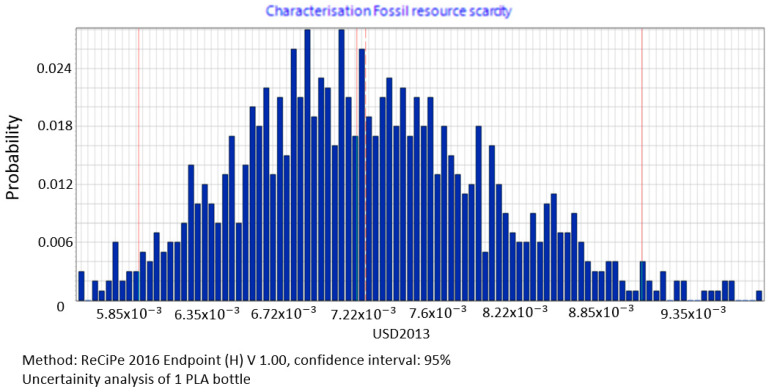
Results of the uncertainty analysis for the category of fossil resources scarcity and its impact on the resources availability.

The unit point data (input) was used to analyze the relevant impact categories, and the results obtained are expressed in units corresponding to each endpoint damage category: human health, ecosystem quality and resources availability. In order to generate the diagrams presented in Figure 8, Figure 9, Figure 10, Figure 11, Figure 12, Figure 13, Figure 14 and Figure 15, categories with a 90% share in damages were used.

Fine particle matter formation was the first significant category in the total impact of biodegradable bottle shaping process on human health. (Figure 8). For data generated in MC simulation, the total level of emissions was found to be 1.61 × 10^−07^ DALY. However, the value of the median was = 1.59 × 10^−07^ DALY, standard deviation = 1.76 × 10^−08^ DALY and coefficient of variation = 10.96%.

The average impact of the manufacture of 1 L PLA bottles on the category of global warming corresponding to human health from 1000 scenarios of input data was 1.27 × 10^−07^ DALY (Figure 9), whereas, the remaining distribution parameters were as follows: median = 1.26 × 10^-7^ DALY, standard deviation = 9.13 × 10^−09^ DALY and the coefficient of variation = 7.20%.

The analysis of all the stages involved in the process of PLA bottle shaping clearly shows that the accumulation of compounds has a high negative impact on the water consumption category (2.74 × 10^−08^ DALY), which adversely affects human health (Figure 10). The median for this is = 3.05 × 10^−08^ DALY, standard deviation = 7.37 × 10^−09^ DALY = 26.8%.

Analyses of stochastic data for all the stages of PLA bottle shaping also covered the emissions of compounds that cause damage in the area of ecosystem quality by contributing to global warming (3.80 × 10^−10^ DALY) (Figure 11). The value of the median is = 3.81 × 10^−10^ species yr, standard deviation= 2.75 × 10^−11^ species yr and the coefficient of variation = 7.20%.

The value of the median of the category of environmental impacts associated with land use (average: 3.32 × 10^−10^) was found to be = 3.17 × 10^−10^ species yr, standard deviation = 4.48 × 10^−11^ species yr and coefficient of variation = 25.1% (Figure 12).

Categories showing significant impact in the ecosystem quality area include water consumption, terrestrial ecosystems, with a mean value of impacts equalling 1.66 × 10^−10^, median = 1.85 × 10^−10^ species yr, a standard deviation of 4.62 × 10^−11^ species yr and a variation of 25.1% (Figure 13).

Figure 14 shows data for accumulated compounds that cause acidification (1.46 × 10^−10^)and their impact on the ecosystem quality. The values of the median = 1.46 × 10^−10^ species yr, standard deviation = 1.32 × 10^−11^ species yr and the coefficient of variation = 9.01%.

Based on the data uncertainty analysis, a histogram of results for the fossil resources scarcity category was drawn up. The mean value of impact in the resource availability area equals 0.0072 USD2013, median = 0.00715 USD2013, standard deviation = 0.00078 USD2013, and variation = 10.8% (Figure 15).

### 3.4. Results of Sensitivity Analysis

**Figure 16:**
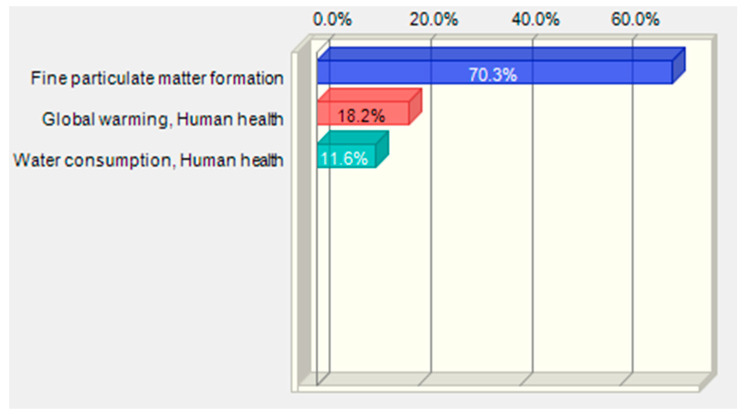
Shares of impact categories in the variance of the total impact of the bottle moulding process in the human health area.

Sensitivity analysis was performed for data represented by impact categories characterising the total impact of the biodegradable bottle preparation lifecycle in three damage areas human health, ecosystem quality and resources availability. Based on the uncertainty distribution of LCA results for relevant impact categories presented in Section 3.3, it was possible to perform the key issue analysis in order to assess the sensitivity of results in the given damage area. The procedure was performed using the MC simulation and the CB software. As Figure 16 and Table 6 show, the category of the formation of fine solid particles represents the greatest share of the variability of the total impact of bottle production on human health. Global warming is in second place. The lowest share in variability is observed in the case of water consumption.

**Figure 17:**
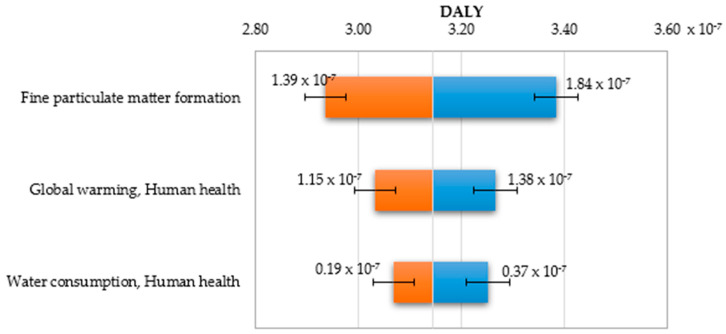
Tornado chart for the forecast of significant categories of impact on human health— error bars show the mean standard error value.

**Figure 18:**
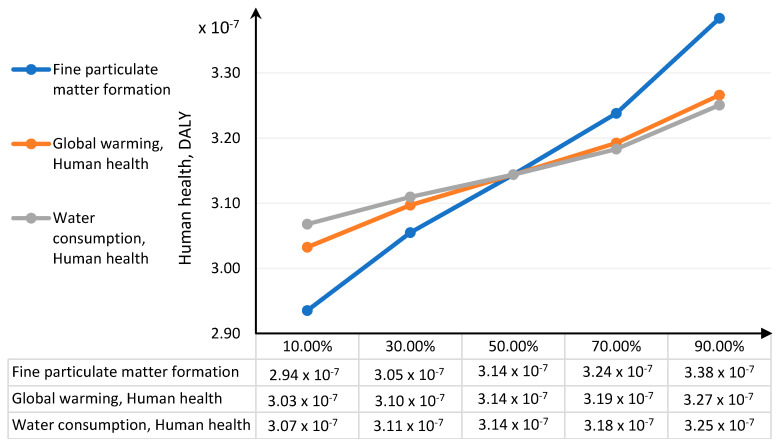
Spider chart of sensitivity for the human health damage category.

The greatest deviations of the human health damage value are observed in the end values of impact categories (Figure 17 and Figure 18). The increase in the fine particulate matter formation has the largest share in the increase of impacts on human health and at the same time a non-accurate estimation of this category will lead to the biggest errors in estimating the value of damages in the area of human health.

**Figure 19:**
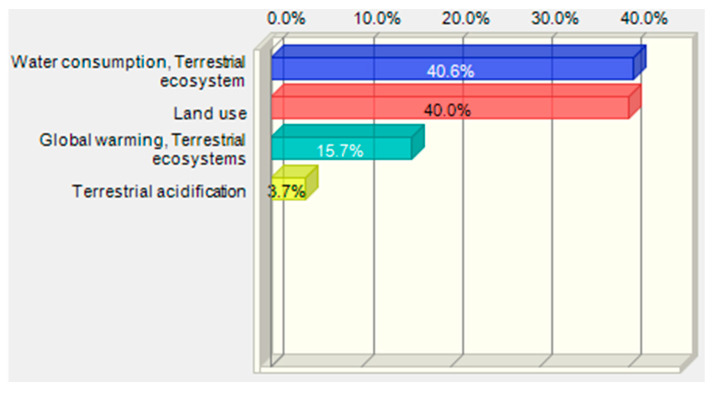
Shares of impact categories in the variance of the total impact of the bottle moulding process in the ecosystem quality area.

For damage category in area ecosystem quality the global water consumption, terrestrial ecosystem represents the greatest share and the next was land use (Table 7, Figure 19).

**Figure 20:**
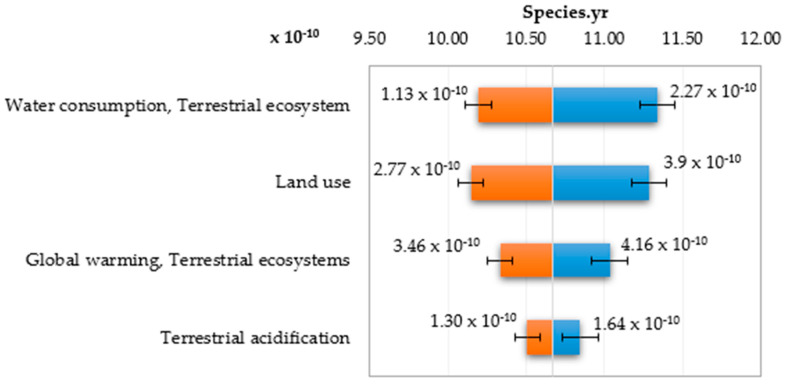
Tornado chart for the forecast of significant categories of impact on ecosystems—error bars show the mean standard error value.

**Figure 21:**
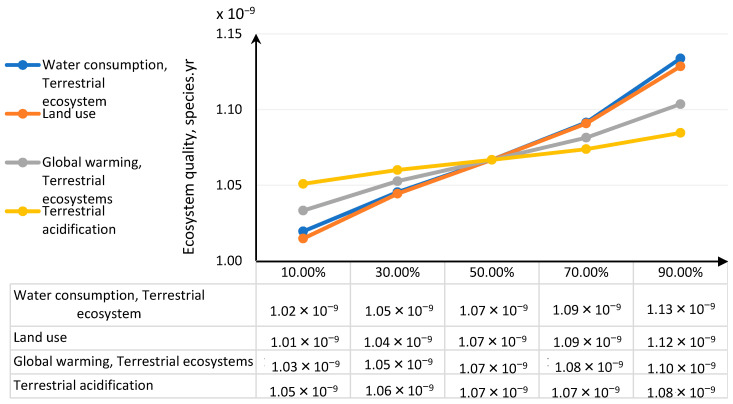
Spider chart of sensitivity for the ecosystem quality damage category.

The greatest deviations of the ecosystem quality damage value are observed in the end values of impact categories (Figure 20 and Figure 21). The increase in the water consumption terrestrial ecosystem category and land use values has the greatest share in the growth of impacts on the ecosystem quality and any imprecise estimation of these categories will lead to greatest errors in the estimation of damage values in the ecosystem quality area.

## 4. Discussion

The focus of this paper is on the evaluation and analysis of LCA results for the bottle moulding process. The results of contribution analysis presented in Section 3.1 show impact categories and stages of the bottle moulding process with the greatest effect on and share in damage categories for LCA results (Figure 4, Figure 5, Figure 6 and Figure 7). When analysing shares of the process stages in midpoint impact categories, as well as in total impacts in damage areas, one has to conclude that pre-forms made of PLA take the first place and are followed by bottle degasifying and pressure shaping as far as negative environmental impacts are concerned (Figure 4, Figure 5, Figure 6 and Figure 7). Stages of the bottle moulding process are closely related to input (inventory) data (Table 4), hence any solutions designed to improve the environmental impact of the bottle shaping process should be directed mainly towards reducing the PLA usage in the production of bottles and reducing the electric energy consumption in the pre-form heating processes (e.g., by means of replacing infrared lamps by less energy-intensive ones), pre-form stretching or cooling. The contribution analysis performed as the first step of the procedure enabled us to determine relevant impact categories to be subjected to the uncertainty and sensitivity analysis.

The procedure proposed in this study required us to perform data quality analysis in order to determine parameters of input data distribution for an uncertainty analysis to be conducted using the MC simulation. In this way, the process of stochastic search for the input parameters distribution was simplified by proposing a triangular distribution as PDF, with deviations from 10% to 50%, depending on the ADUI result. Determining the level of deviations is one of the elements of this search and affects the simulated values of expected results. The input data collected by us were characterised by high quality (ADQI = 5) and precision, as they were obtained directly form a manufacturing company. In the uncertainty analysis, a deviation of ±10% was used, according to the triangular distribution. The analysis of the diagrams presented in Figure 8, Figure 9 and Figure 10 shows that the category associated with the use of water resources (Figure 10) has the largest variability range (deviation 26.8%), thus being the most critical input variable affecting human health throughout the life cycle of the product considered. Yet in the category of harmful impact on the ecosystem, the widest variability interval was found for the category of land use (deviation 25.1%, Figure 12) and water consumption connected, terrestrial ecosystems (deviation 25.1%, Figure 13). In the resource availability damage category, the variability of the prevailing impact category of fossil resources scarcity was 10.8% (Figure 15). The pre-form material was a key determinant of this category value. Input data deviations for the above-listed categories in the range of 10% translated into a significant variability of results, therefore the precision and accuracy of the input data will be of key importance to the values of these categories. With imprecise, low quality data, the result is least certain in these categories. As far as the final result is concerned, it is most important to measure the mass of the pre-form to be used in the blowing process and the consumption of water at the bottle cooling stage with greatest precision.

The results distribution characteristics obtained for significant impact categories as a result of the uncertainty analysis (i.e., mean, standard value) were used for performing the key issue analysis using the MC simulation and the CB software. The analysis enabled us to identify the impact categories with the greatest shares in the uncertainty of the final result in the damage category. The fine particulate matter formation category had the greatest share (69.5%) in the human health damage category variance. Depending on changes in this impact category, the total impact in the human health area will be subject to most serious changes. In the ecosystem quality area, water consumption had the greatest share as regards terrestrial ecosystem and land use. As regards these impact categories, the greatest changes should be also expected wherever even a slight decrease in the input parameters value takes place in order to improve the environmental impact of the PLA bottle shaping process. In the group of impact categories referred to above, low quality input data would introduce significant uncertainty of the final impact results in damage categories. The final impact result in the damage category is most sensitive to changes in the impact categories referred to above.

**Figure 22:**
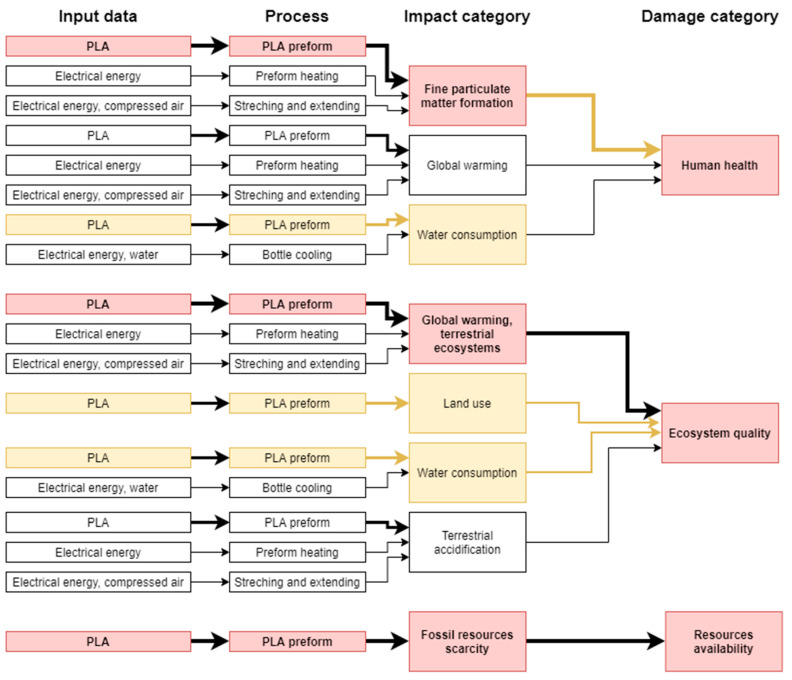
Paths of key links between input data and impact and damage categories. The figure presents input data for only significant stages of the PLA bottle shaping process and relevant impact categories. Bold arrows represent inputs with the greatest share in the process or category. Chains marked in red are those with the greatest share in the damage category, while yellow chains bring the greatest uncertainty with respect to the final result of the impact and damage category.

The method proposed for the evaluation of data quality and uncertainty permits to easily identify key parameters that affect the final result of LCA (without the need to calculate complex endpoint coefficients and parameters of the results distribution shape). A single procedure combines three types of analyses: the quality of data, uncertainty of results and sensitivity with respect to input (inventory) data and impact categories, thereby enabling one to determine qualitative and quantitative relationships between input data, impact categories and impact areas, as presented in Figure 22. Clearly, although some categories show a relatively high uncertainty, they will not have any significant effect on the final result, e.g., the water consumption category, despite its high uncertainty, will not have any serious impact on the human health damage category. But then, in the case of the ecosystem quality damage category, two impact categories will cause the greatest uncertainty of results: water consumption and land use, although they do not represent the greatest shares in this damage category.

In practice, data uncertainty assessment procedures come down to one type of analysis, e.g., the semi-quantitative DQI approach [20], stochastic modelling with the use of the MC simulation [22,48], sensitivity analysis [10,65]. Combinations of several methods are not common, due to the significant input of labour and time required and a great number of data than need to be entered and collected [62,72]. However, the method proposed here, is based on inventory outcomes and each step produces a set of inputs for the next stage, thereby providing a network of links between input data and impact categories, as well as damage categories (Figure 22).

The types of variables distribution used in these studies (triangle distribution for input data and lognormal distribution for impact categories), as well as range endpoints, are not without effect on the value of results obtained either. Using other distributions might lead to small differences in the uncertainty results, as shown in [20,38,65,72]. It should be pointed out that the input data distribution is based on the semi-quantitative DQI approach result and it is largely the quality of data that will determine results of any further stages.

## 5. Conclusions

We have achieved the objective of the paper by proposing a method for the evaluation of uncertainty and precision of LCA results based on the DQI semi-quantitative approach, stochastic modelling and sensitivity analysis. The method permits to simply determine dependencies between the precision of input data and impact and damage categories, by identifying key elements that affect the LCA result. The strong point of the methodology used is that not much data need to be collected for the analyses.

The procedure identified input data of the PLA bottle shaping process, as well as sensitive impact categories. The material used for producing the pre-form (PLA) represents the input which has the greatest effect on the result of environmental impacts of the PLA bottle shaping process. The fact that the mass of material is reduced during the bottle shaping process is the greatest contributor to the reduction of environmental impacts. At the same time, the accuracy and precision of the PLA mass estimation will be the key element affecting the final result uncertainty, while the accuracy of water and electric energy consumption estimations will be less important. Impact categories with the greatest uncertainty include water consumption with respect to human health and land use and water consumption with respect to ecosystem quality. On the other hand, the uncertainty and development of the final result value in the human health damage category depend mostly on the fine particulate matter formation category and in the ecosystem quality damage category—on water consumption and land use. In the area of resources availability, the impact category of fossil resources scarcity is mainly responsible for both the uncertainty and value of results, its values being determined by the value of PLA pre-form material used in the process. In the context of improving the environmental balance of the bottle shaping process, the consumption of energy in the processes of heating, stretching and cooling should be brought down and water consumption should be reduced along with the pre-form material consumption.

## Figures and Tables

**Table 1:** Pedigree matrix [56,60].

DQI	DQI = 5.0	DQI = 4.0	DQI = 3.0	DQI = 2.0	DQI = 1.0
**R**	Data verified and based on measurements	Verified data based partly on assumptions, or unverified based on measurements	Unverified data based partly on assumptions	Precise estimation	Inaccurate estimation
**C**	Representative data taken from the appropriate sample and time range	Data collected from a smaller sample, but within a reasonable period of time	Data taken from an appropriate sample, but not in the right time range	Representative data, but from a very small sample	Data not representative from a very small sample or it is unknown
**TR**	Deviation up to 3 years	Deviation up to 6 years	Deviation up to 10 years	Deviation up to 15 years	Deviation over 15 years or data age unknown
**GS**	Local scope (or other area envisaged for the purpose of the study)	National scope	Continental range	Global scope	Data of unknown origin
**TS**	Data on the analyzed process and enterprise	Data on the analyzed process and technology, but from a different source	Data on the analyzed process, but with different technology	Data on similar processes/products with the same technology	Data on similar processes/products with different technology

R—reliability, C—completeness, TR—time range, GS—geographical scope, TS—technological scope.

**Table 2:** Aggregated data quality indicator (ADQI) values and deviation levels [54,60].

ADQI	Deviation (%)	ADQI	Deviation (%)	ADQI	Deviation (%)	ADQI	Deviation (%)
5.0	10	4.0	20	3.0	30	2.0	40
4.8	12	3.8	22	2.8	32	1.8	42
4.6	14	3.6	24	2.6	34	1.6	44
4.4	16	3.4	26	2.4	36	1.4	46
4.2	18	3.2	28	2.2	38	1.2	48
						1.0	50

**Table 3:** Sample electricity test results specified for the preform stretching and extending process.

Medium	Quantity	Distribution	Minimum	Maximum	DQI
Electricity	6.95 kWh	Triangular	6.26	7.64	5

**Table 4:** Results LCI [55].

Technological Operations	Ecoinvent Activity	Amount
**Raw material acquisition**		
PLA preform mass	Polylactide, granulate {GLO} market for/Alloc Def, S	18.24 g
Electrical energy	Electricity, medium voltage {PL} market for/Alloc Def, S	0.368 kWh
**Preform heating**		
Electrical energy (infrared lamp 100 kW)	Electricity, medium voltage {PL} market for/Alloc Def, S	3.2 kWh
Electrical energy (infrared lamps 200 kW)		6.4 kWh
Electrical energy (supply chain)		0.16 kWh
**Preform stretching and extending**		
Electrical energy	Electricity, medium voltage {PL} market for/Alloc Def, S	6.95 kWh
Compressed air	Compressed air, 1000 kPa gauge {RER} compressed air production/Alloc Def U	0.0016 kg/m^3^
**Preform pressure shaping**		
Electrical energy	Electricity, medium voltage {PL} market for/Alloc Def, S	5.66 kWh
**Bottle degasifying**		
Electrical energy	Electricity, medium voltage {PL} market for/Alloc Def, S	1.01 kWh
**Bottle cooling**		
Electrical energy	Electricity, medium voltage {PL} market for/Alloc Def, S	0.71 kWh
Water in a closed circulation	Tap water {Europe without Switzerland} market for/Alloc Def, S	2.4 m^3^

**Table 5:** Values of the minimum and maximum for inventory elements of the process of polylactide bottle manufacturing.

Element LCI	Value		ADQI	Deviation (%)	Min.	Max.	Distribution
**Raw material acquisition**							
Electrical energy (three motors of the carousel)	0.368	kWh	5	10	0.33	0.404	Triangular
PLA preform mass	18.24	g	5	10	16.42	20.064	
**Preform heating**							
Electrical energy (infrared lamp 100 kW)	3.2	kWh	5	10	2.88	3.52	Triangular
Electrical energy (infrared lamps 200 kW)	6.4	kWh	5	10	5.76	7.04	
Electrical energy (supply chain)	0.16	kWh	5	10	0.14	0.176	
**Preform stretching and extending**							
Electrical energy	6.95	kWh	5	10	6.26	7.64	Triangular
Compressed air	0.0016	kg/m^3^	5	10	0.00144	0.00176	
**Preform pressure shaping**							
Electrical energy	5.66	kWh	5	10	5.09	6.22	Triangular
**Bottle degasifying**							
Electrical energy	1.01	kWh	5	10	0.91	1.11	Triangular
**Bottle cooling**							
Electrical energy	0.71	kWh	5	10	0.64	0.78	Triangular
Water in closed circulation	2.4	m^3^	5	10	2.16	2.64	

**Table 6:** Results of the Monte Carlo (MC) simulation performed in Crystal Ball (CB) for the tornado-type sensitivity analysis of the significant categories of impact on human health.

	Human Health, 10^−7^ DALY				Input, 10^−7^ DALY		
Input Data	Lower Limit	Upper Limit	Range	Deviation Explained ^1^	Lower Limit	Upper Limit	Base Case ^2^
Fine particulate matter formation	2.94	3.39	0.45	70.28%	1.39	1.84	1.60
Global warming, human health	3.03	3.27	0.23	88.46%	1.15	1.38	1.26
Water consumption, human health	3.07	3.25	0.18	100.00%	0.19	0.37	0.26

^1^ The explained deviation is cumulative, ^2^ The basic case for calculations in CB was the median value.

**Table 7:** Results of the MC simulation performed in CB for the tornado-type sensitivity analysis of the significant categories of impact on ecosystem quality.

	Human Health, 10^−9^ DALY				Input, 10^−10^ DALY		
Input Data	Lower Limit	Upper Limit	Range	Deviation Explained ^1^	Lower Limit	Upper Limit	Base Case ^2^
Water consumption, Terrestrial ecosystem	1.02	1.13	0.114	40.69%	1.13	2.27	1.60
Land use	1.01	1.12	0.113	81.06%	2.77	3.90	3.29
Global warming, Terrestrial ecosystems	1.03	1.10	0.070	96.46	3.46	4.16	3.79
Terrestrial acidification	1.05	1.08	0.033	100.00%	1.30	1.64	1.46

^1^ The explained deviation is cumulative, ^2^ The basic case for calculations in CB was the median value.

## References

[1] International Organization for Standardization (2006). ISO 14040:2006—Environmental Management—Life Cycle Assessment—Principles and Framework.

[2] International Organization for Standardization (2006). ISO 14044:2006—Environmental Management—Life Cycle Assessment—Requirements and Guidelines.

[3] Mannheim V., Fehér Z.S., Siménfalvi Z. (2019). Innovative solutions for the building industry to improve sustainability performance with Life Cycle Assessment modelling. Solutions for Sustainable Development.

[4] Tomporowski A., Piasecka I., Flizikowski J., Kasner R., Kruszelnicka W., Mroziński A., Bieliński K. (2018). Comparison Analysis of Blade Life Cycles of Land-Based and Offshore Wind Power Plants. Pol. Marit. Res..

[5] Huijbregts M.A.J. (1998). Application of uncertainty and variability in LCA. Int. J. Life Cycle Assess..

[6] Heijungs R., Huijbregts M.A.J. A Review of Approaches to Treat Uncertainty in LCA. Proceedings of the International Congress on Environmental Modelling and Software.

[7] Magrassi F., Del Borghi A., Gallo M., Strazza C., Robba M. (2016). Optimal Planning of Sustainable Buildings: Integration of Life Cycle Assessment and Optimization in a Decision Support System (DSS). Energies.

[8] Lloyd S.M., Ries R. (2007). Characterizing, Propagating, and Analyzing Uncertainty in Life-Cycle Assessment: A Survey of Quantitative Approaches. J. Ind. Ecol..

[9] Pomponi F., D’Amico B., Moncaster A.M. (2017). A Method to Facilitate Uncertainty Analysis in LCAs of Buildings. Energies.

[10] Heijungs R. (2010). Sensitivity coefficients for matrix-based LCA. Int. J. Life Cycle Assess..

[11] Heijungs R., Frischknecht R. (2005). Representing Statistical Distributions for Uncertain Parameters in LCA. Relationships between mathematical forms, their representation in EcoSpold, and their representation in CMLCA (7 pp). Int. J. Life Cycle Assess..

[12] Maurice B., Frischknecht R., Coelho-Schwirtz V., Hungerbühler K. (2000). Uncertainty analysis in life cycle inventory. Application to the production of electricity with French coal power plants. J. Clean. Prod..

[13] Heijungs R. (1996). Identification of key issues for further investigation in improving the reliability of life-cycle assessments. J. Clean. Prod..

[14] Wang E., Shen Z., Neal J., Shi J., Berryman C., Schwer A. (2012). An AHP-weighted aggregated data quality indicator (AWADQI) approach for estimating embodied energy of building materials. Int. J. Life Cycle Assess..

[15] Canter K.G., Kennedy D.J., Montgomery D.C., Keats J.B., Carlyle W.M. (2002). Screening stochastic Life Cycle assessment inventory models. Int. J. Life Cycle Assess..

[16] Frischknecht R., Jungbluth N., Althaus H.J., Doka G., Dones R., Heck T., Hellweg S., Hischier R., Nemecek T., Rebitzer G. (2005). The ecoinvent Database: Overview and Methodological Framework (7 pp). Int. J. Life Cycle Assess..

[17] Kennedy D.J., Montgomery D.C., Rollier D.A., Keats J.B. (1997). Data Quality: Assessing Input Data Uncertainty in Life Cycle Assessment Inventory Models. Int. J. Life Cycle Assess..

[18] Weidema B.P., Wesnæs M.S. (1996). Data quality management for life cycle inventories—An example of using data quality indicators. J. Clean. Prod..

[19] Weidema B.P. (1998). Multi-user test of the data quality matrix for product life cycle inventory data. Int. J. Life Cycle Assess..

[20] Baek C.Y., Tahara K., Park K.H. (2018). Parameter Uncertainty Analysis of the Life Cycle Inventory Database: Application to Greenhouse Gas Emissions from Brown Rice Production in IDEA. Sustainability.

[21] Muller S., Lesage P., Ciroth A., Mutel C., Weidema B.P., Samson R. (2016). The application of the pedigree approach to the distributions foreseen in ecoinvent v3. Int. J. Life Cycle Assess..

[22] Sonnemann G.W., Schuhmacher M., Castells F. (2003). Uncertainty assessment by a Monte Carlo simulation in a life cycle inventory of electricity produced by a waste incinerator. J. Clean. Prod..

[23] International Organization for Standarization (2003). ISO/TR 14047:2003—Environmental Management—Life Cycle Impact Assessment—Examples of application of ISO 14042.

[24] International Organization for Standarization (2002). ISO/TS 14048:2002—Environmental Management—Life Cycle Assessment—Data Documentation Format.

[25] Hedbrant J., Sörme L. (2001). Data Vagueness and Uncertainties in Urban Heavy-Metal Data Collection. Water Air Soil Pollut. Focus.

[26] Lewandowska A. (2011). Environmental life cycle assessment as a tool for identification and assessment of environmental aspects in environmental management systems (EMS) part 1: Methodology. Int. J. Life Cycle Assess..

[27] Shen L., Worrell E., Patel M.K. (2010). Open-loop recycling: A LCA case study of PET bottle-to-fibre recycling. Resour. Conserv. Recycl..

[28] Lewandowska A., Matuszak-Flejszman A. (2014). Eco-design as a normative element of Environmental Management Systems—The context of the revised ISO 14001:2015. Int. J. Life Cycle Assess..

[29] Steen B. (1997). On uncertainty and sensitivity of LCA-based priority setting. J. Clean. Prod..

[30] Kowalski Z., Kulczycka J. (2005). Ocena cyklu życia LCA jako podstawowy czynnik oceny czystszych produkcji. Odzysk odpadów—Technologie i możliwości. Materiały Konfrerencji, Waste Recycling.

[31] Lewandowska A., Foltynowicz Z. (2007). Eco-Design as a New Trend in Packing Materials Production. Zesz. Nauk. Akad. Ekon. Pozn..

[32] Heijungs R., Suh S. (2002). The Computational Structure of Life Cycle Assessment.

[33] André J.C.S., Lopes D.R. (2012). On the use of possibility theory in uncertainty analysis of life cycle inventory. Int. J. Life Cycle Assess..

[34] Benetto E., Dujet C., Rousseaux P. (2008). Integrating fuzzy multicriteria analysis and uncertainty evaluation in life cycle assessment. Environ. Model. Softw..

[35] Egilmez G., Gumus S., Kucukvar M., Tatari O. (2016). A fuzzy data envelopment analysis framework for dealing with uncertainty impacts of input–output life cycle assessment models on eco-efficiency assessment. J. Clean. Prod..

[36] Von Bahr B., Steen B. (2004). Reducing epistemological uncertainty in life cycle inventory. J. Clean. Prod..

[37] Lasvaux S., Schiopu N., Habert G., Chevalier J., Peuportier B. (2014). Influence of simplification of life cycle inventories on the accuracy of impact assessment: Application to construction products. J. Clean. Prod..

[38] Baek C.Y., Park K.H., Tahara K., Chun Y.Y. (2017). Data Quality Assessment of the Uncertainty Analysis Applied to the Greenhouse Gas Emissions of a Dairy Cow System. Sustainability.

[39] Chou J.S., Yeh K.C. (2015). Life cycle carbon dioxide emissions simulation and environmental cost analysis for building construction. J. Clean. Prod..

[40] Niero M., Pizzol M., Bruun H.G., Thomsen M. (2014). Comparative life cycle assessment of wastewater treatment in Denmark including sensitivity and uncertainty analysis. J. Clean. Prod..

[41] Geisler G., Hellweg S., Hungerbühler K. (2005). Uncertainty Analysis in Life Cycle Assessment (LCA): Case Study on Plant-Protection Products and Implications for Decision Making (9 pp + 3 pp). Int. J. Life Cycle Assess..

[42] Hong J., Shen G.Q., Peng Y., Feng Y., Mao C. (2016). Uncertainty analysis for measuring greenhouse gas emissions in the building construction phase: A case study in China. J. Clean. Prod..

[43] Chevalier J.L., Téno J.F.L. (1996). Life cycle analysis with ill-defined data and its application to building products. Int. J. Life Cycle Assess..

[44] Reap J., Roman F., Duncan S., Bras B. (2008). A survey of unresolved problems in life cycle assessment. Int. J. Life Cycle Assess..

[45] Miller S.A., Moysey S., Sharp B., Alfaro J. (2013). A Stochastic Approach to Model Dynamic Systems in Life Cycle Assessment. J. Ind. Ecol..

[46] Thiel C.L., Campion N., Landis A.E., Jones A.K., Schaefer L.A., Bilec M.M. (2013). A Materials Life Cycle Assessment of a Net-Zero Energy Building. Energies.

[47] Cambridge University Built Environment Sustainability (CUBES) Risk and Uncertainty in Embodied Carbon Assessment. Proceedings of the Cambridge University Built Environment Sustainability (CUBES) Embodied Carbon Symposium 2016.

[48] Peters G.P. (2006). Efficient algorithms for Life Cycle Assessment, Input-Output Analysis, and Monte-Carlo Analysis. Int. J. Life Cycle Assess..

[49] Heijungs R., Lenzen M. (2014). Error propagation methods for LCA—A comparison. Int. J. Life Cycle Assess..

[50] Heijungs R., Tan R.R. (2010). Rigorous proof of fuzzy error propagation with matrix-based LCI. Int. J. Life Cycle Assess..

[51] Imbeault-Tétreault H., Jolliet O., Deschênes L., Rosenbaum R.K. (2013). Analytical Propagation of Uncertainty in Life Cycle Assessment Using Matrix Formulation. J. Ind. Ecol..

[52] Ciroth A., Fleischer G., Steinbach J. (2004). Uncertainty calculation in life cycle assessments. Int. J. Life Cycle Assess..

[53] Piotrowska K., Kruszelnicka W., Baldowska-Witos P., Kasner R., Rudnicki J., Tomporowski A., Flizikowski J., Opielak M. (2019). Assessment of the Environmental Impact of a Car Tire throughout Its Lifecycle Using the LCA Method. Materials.

[54] Bałdowska-Witos P., Kruszelnicka W., Kasner R., Tomporowski A., Flizikowski J., Mrozinski A. (2019). Impact of the plastic bottle production on the natural environment. Part 2. Analysis of data uncertainty in the assessment of the life cycle of plastic beverage bottles using the Monte Carlo technique. Przem. Chem..

[55] Bałdowska-Witos P., Kruszelnicka W., Kasner R., Tomporowski A., Flizikowski J., Kłos Z., Piotrowska K., Markowska K. (2020). Application of LCA Method for Assessment of Environmental Impacts of a Polylactide (PLA) Bottle Shaping. Polymers.

[56] Edelen A., Ingwersen W. (2016). Guidance on Data Quality Assessment for Life Cycle Inventory Data.

[57] Bulle C., Margni M., Patouillard L., Boulay A.M., Bourgault G., De Bruille V., Cao V., Hauschild M., Henderson A., Humbert S. (2019). IMPACT World+: A globally regionalized life cycle impact assessment method. Int. J. Life Cycle Assess..

[58] Fregonara E., Ferrando D.G., Pattono S. (2018). Economic–Environmental Sustainability in Building Projects: Introducing Risk and Uncertainty in LCCE and LCCA. Sustainability.

[59] Ogilvie S., Collins M., Aumônier S. (2004). Life Cycle Assessment of the Management Options for Waste Tyres.

[60] Lewandowska A. (2006). Środowiskowa Ocena Cyklu Życia Produktu na Przykładzie Wybranych Typów Pomp Przemysłowych.

[61] International Organization for Standardization (2002). ISO 14042:2002—Environmental Management—Life Cycle Assessment—Life Cycle Impact Assessment.

[62] Bieda B. (2014). Metoda Monte Carlo w Ocenie Niepewności w Stochastycznej Analizie w Przemyśle Stalowniczym i Inżynierii Środowiska.

[63] Kennedy P. (2003). A Guide to Econometrics.

[64] Saltelli A., Annoni P. (2010). How to avoid a perfunctory sensitivity analysis. Environ. Model. Softw..

[65] Groen E.A., Bokkers E.A.M., Heijungs R., de Boer I.J.M. (2017). Methods for global sensitivity analysis in life cycle assessment. Int. J. Life Cycle Assess..

[66] Groen E.A., Heijungs R., Bokkers E.A.M., de Boer I.J.M. (2014). Sensitivity analysis in life cycle assessment. Proceedings of the 9th International Conference on Life Cycle Assessment in the Agri-Food Sector.

[67] Heijungs R. The use of matrix perturbation theory for addressing sensitivity and uncertainty issues in LCA. Proceedings of the Fifth International Conference on EcoBalance—Practical Tools and Thoughtful Principles for Sustainability.

[68] Heijungs R., Suh S., Kleijn R. (2005). Numerical Approaches to Life Cycle Interpretation—The case of the Ecoinvent’96 database (10 pp). Int. J. Life Cycle Assess..

[69] Huibregts M.A.J., Steinmann Z.J.N., Elshout P.M.F., Stam G., Verones F., Vieira M.D.M., Hollander A., Zijp M., Van Z. (2016). ReCiPe 2016 A Harmonized Life Cycle Impact Assessment Method at Midpoint and Endpoint Level Report I: Characterization.

[70] Huijbregts M.A.J., Steinmann Z.J.N., Elshout P.M.F., Stam G., Verones F., Vieira M., Zijp M., Hollander A., van Zelm R. (2017). ReCiPe2016: A harmonised life cycle impact assessment method at midpoint and endpoint level. Int. J. Life Cycle Assess..

[71] Bakshi B.R. (2019). Sustainable Engineering: Principles and Practice.

[72] Mutel C.L., de Baan L., Hellweg S. (2013). Two-Step Sensitivity Testing of Parametrized and Regionalized Life Cycle Assessments: Methodology and Case Study. Environ. Sci. Technol..

